# Methicillin-resistant *Staphylococcus aureus* in Dutch Soccer Team

**DOI:** 10.3201/eid1210.060387

**Published:** 2006-10

**Authors:** Xander W. Huijsdens, Ans M.C. van Lier, Eric van Kregten, Liesbeth Verhoef, Marga G. van Santen-Verheuvel, Emile Spalburg, Wim J.B. Wannet

**Affiliations:** * National Institute for Public Health and the Environment, Bilthoven, the Netherlands;; † De Gemeenschappelijke Gezondheidsdienst Eemland, Amersfoort, the Netherlands;; ‡ Meander Medical Centre, Amersfoort, the Netherlands;; § Saltro, Utrecht, the Netherlands

**Keywords:** Methicillin resistance, *Staphylococcus aureus*, community-acquired infections, Panton-Valentine leukocidin, sports, soccer, dispatch

## Abstract

An outbreak of community-acquired methicillin-resistant *Staphylococcus aureus* occurred among members and close contacts of a soccer team. Typing of the isolates showed the outbreak was caused by the well-known European ST80-IV strain. To our knowledge, this is the first report of an outbreak of this strain among members of a sports team.

Community-acquired methicillin-resistant *Staphylococcus aureus* (CA-MRSA) is emerging as a cause of skin and soft tissue infections. CA-MRSA differs from hospital-associated MRSA in several ways (*1*). Most CA-MRSA isolates contain the virulence factor Panton-Valentine leukocidin (PVL) and carry staphylococcal cassette chromosome (SCC) *mec* type IV or V (*2*). In the Netherlands the prevalence of MRSA is still low (≈1%) (*3*), and ≈10 % of all MRSA isolates carry the genes for PVL (*4*).

Several reports have been published about CA-MRSA outbreaks among sports team members, communities, prisoners, men who have sex with men, military personnel, and drug users (*5*). Most of these cases occurred in the United States and were caused by USA300 MRSA sequence type (ST) 8 strains. In Europe the ST80-MRSA-IV strain is common (*4**,**6*) but has not been reported to have caused an outbreak in sports teams. A CA-MRSA outbreak in a British rugby team has been reported (*7*), but given the reported phage type, this strain was not ST80-MRSA-IV. So far, CA-MRSA outbreaks have usually occurred in intensive contact sports such as football (*8**–**10*), rugby (*7*), and wrestling (*11*).

We report on the results of the screening that was conducted after MRSA was isolated from a boil on a Dutch soccer player and, subsequently, soft-tissue infections developed on other members of the soccer team.

## The Study

Starting in June 2005, several players of a Dutch soccer team, consisting of ≈35 members, noted soft-tissue infections. In October 2005, the municipal health service received a report of a patient, a member of this soccer team, who had been hospitalized for an abscess resulting from MRSA. Other members of the team had skin infections as well, and screening was started.

A case was defined as a patient who had a culture-confirmed MRSA infection during the outbreak period October 2005 through January 2006. Healthcare staff obtained specimens by swabbing the patients' nose, throat, or wound. A total of 56 persons were screened: 42 members of the soccer club and 14 of their roommates. The 42 members consisted of soccer players, coaches, and people who used the same training facilities, locker room, and showers. The roommates screened were all those who lived with an MRSA-positive player and those who lived with an MRSA-negative player but had skin infections. Of the 56 persons screened, we identified an MRSA infection in 11 persons: 9 soccer players and 2 roommates (Table). Most infections lasted for several weeks. For all players who had soft tissue infections, MRSA was diagnosed. Among those in whom infections did not develop, no carriage of MRSA was found. One roommate had an abscess in the armpit, but the soccer player she lived with was MRSA negative. The mean age of the patients was 31 years (range 18–43). No histories of travel were reported.

**Table:** Characteristics of MRSA infections, soccer players and their contacts, the Netherlands, 2005*

No.	Patient	Age, y	Type of infection	Site of infection	MRSA carriage in nose	MRSA carriage in GI tract	Recurring infection
1	Player†	34	Abscesses	NA	NA	NT	NA
2	Player	30	Boil	Leg	Yes	NT	Yes
3	Player	43	NS	Arm	Yes	Yes	Yes
4	Player	33	NS	Forearm	Yes	Yes	Yes
5	Player	33	Boil	Leg	No	NT	No
6	Player	34	Boil	Knee	No	NT	No
7	Player	22	Boil	Leg	Yes	NT	Yes
8	Roommate of no.4	31	NS	Face	NA	No	NA
9	Player	18	NS	Buttock	Yes	Yes	No
10	Player	20	NS	Heel	Yes	NT	No
11	Roommate of an MRSA-negative player	39	Abscesses	Armpit	No	Yes	No
12	Opponent	33	Boil	Leg	NA	NT	No

*MRSA, methicillin-resistant *Staphylococcus aureus;* GI, gastrointestinal; NA, not available; NT, not tested; NS, not specified. †This player was the index case-patient and was hospitalized.

To prevent further MRSA transmission, on October 28 the soccer club was instructed to increase hand hygiene, not share personal items, use liquid soap and disposable towels, put a towel on the bench before sitting, increase frequency of cleaning the facilities, and provide more ventilation to the locker room and showers. All patients were treated with cotrimoxazole for 1 week or, if needed, longer until wound infections were healed. Seven patients remained MRSA positive or had recurring wound infections. Perineum cultures from 3 patients showed carriage of MRSA in the gastrointestinal tract, and rifampicin was added to cotrimoxazole for 1 week or, if needed, longer until wounds were healed. Patients were advised to use clean bedding and clothing every day. Furthermore, patients used chlorohexidine, gluconate scrub, povidone iodine, and mupirocin ointment for 5 days. A patient was declared MRSA free after 3 cultures, taken at 1-week intervals, were MRSA negative. One patient, who also had eczema, remained MRSA positive.

In November 2005, MRSA was isolated from a 33-year-old soccer player with a boil, who was a member of a neighboring team. This soccer player had competed against the team with the MRSA-positive players on October 8, before MRSA-positive results were known and hygiene measures recommended. Immediately, this player's team was screened for MRSA; all other players were MRSA negative. The MRSA isolate from this player was included in the analysis.

To characterize the MRSA strains, the following typing methods were used: pulsed-field gel electrophoresis (PFGE), staphylococcal protein A (*spa*) typing, multilocus sequence typing, SCC*mec* typing, and PCR of PVL genes (*LukS*-*LukF*). All MRSA isolates were identical and identified as the European CA-MRSA ST80-MRSA-IV strain. All strains were PFGE type 28 (according to the Dutch PFGE classification system), *spa* type t044, ST80, SCC*mec* IV, and PVL positive. All MRSA isolates had identical susceptibility patterns; they were resistant to oxacillin (and thus to all β-lactam antimicrobial drugs), tetracycline, and fusidic acid. They were susceptible to rifampicin, ciprofloxacin, gentamicin, erythromycin, clindamycin, vancomycin, teicoplanin, and cotrimoxazole.

## Conclusions

This study shows transmission of the CA-MRSA ST80-IV strain among members of a Dutch soccer team. Transmission apparently occurred not only between members of this team but also to a competing soccer team. Transmission of the USA300 strain between members of different teams was previously described for football teams (*8*). We cannot rule out the possibility of an independent colonization of the ST80-MRSA-IV strain in the competing team, but the 2 teams competed against each other during the period when the first symptoms were noted by members of the first team. Because soccer involves much less contact than football, rugby, or wrestling, MRSA transmission may not necessarily have been caused by skin-to-skin contact but could also have occurred by sharing equipment or personal items. This possibility has also been suggested in a report about a CA-MRSA outbreak among sports participants (*12*).

To our knowledge, this is the first report of an outbreak of the CA-MRSA ST80-IV strain in a sports team. As with the USA300 strain, more outbreaks of CA-MRSA ST80-IV are likely. Therefore, to identify and control an outbreak as early as possible, sports physicians and coaching staff should be alerted and informed about CA-MRSA.
